# A recombinant attenuated candidate vaccine that efficiently and persistently protects against bovine leukemia virus in herds

**DOI:** 10.1186/1742-4690-11-S1-O10

**Published:** 2014-01-07

**Authors:** Sabrina M Rodríguez, Gerónimo Gutiérrez, Nicolas Gillet, Karina Trono, Luc Willems

**Affiliations:** 1 Molecular and Cellular Epigenetics, Interdisciplinary Cluster for Applied Genoproteomics (GIGA), University of Liège (ULg), Liège, Belgium; 2 Molecular and Cellular Biology, Gembloux Agro-Bio Tech, University of Liège (ULg), Gembloux, Belgium; 3 Instituto de Virología, CICVyA, Instituto Nacional de Tecnología Agropecuaria, Castelar, Argentina

## 

There currently is no efficient vaccine that protects against bovine leukemia virus (BLV) infection. We now propose a novel approach based on the use of a recombinant live-attenuated BLV provirus. The rationale behind this strategy relies on the deletion of genes required to induce pathogenesis maintaining the integrity of those involved in infectivity. We have identified a BLV attenuated provirus that is infectious but replicates at reduced levels in cattle. The attenuated strain elicits a strong anti-BLV immune response and does not spread to uninfected sentinels maintained during 3 years in the same herd supporting biosafety of the vaccine. Passive antibodies are transmitted to newborn calves via maternal colostrum and persist during several months. Nevertheless, the BLV attenuated provirus does not transmit from cows to calves. Finally, vaccinated animals resist challenge by a wild-type BLV virus. In summary, we have identified a safe BLV attenuated provirus with impaired transmissibility that efficiently protects against infection in herd conditions.

